# LppA is a novel plasminogen receptor of *Mycoplasma bovis* that contributes to adhesion by binding the host extracellular matrix and Annexin A2

**DOI:** 10.1186/s13567-023-01242-1

**Published:** 2023-11-17

**Authors:** Shuang Liu, Zhangcheng Li, Shimei Lan, Huafang Hao, Xiangrui Jin, Jinjia Liang, Ahmed Adel Baz, Xinmin Yan, Pengcheng Gao, Shengli Chen, Yuefeng Chu

**Affiliations:** 1 State Key Laboratory for Animal Disease Control and Prevention, College of Veterinary Medicine, Lanzhou University, Lanzhou Veterinary Research Institute, Chinese Academy of Agricultural Sciences, Lanzhou, 730000 China; 2 Gansu Province Research Center for Basic Disciplines of Pathogen Biology, Lanzhou, 730046 China; 3 Key Laboratory of Veterinary Etilogoical Biology, Key Laboratory of Ruminant Disease Prevention and Control (West), Ministry of Agricultural and Rural Affairs, Lanzhou, 730046 China

**Keywords:** *Mycoplasma bovis*, LppA, extracellular matrix, plasminogen, Annexin A2, adhesion

## Abstract

*Mycoplasma bovis* is responsible for various inflammatory diseases in cattle. The prevention and control of *M. bovis* are complicated by the absence of effective vaccines and the emergence of multidrug-resistant strains, resulting in substantial economic losses worldwide in the cattle industry. Lipoproteins, vital components of the Mycoplasmas cell membrane, are deemed potent antigens for eliciting immune responses in the host upon infection. However, the functions of lipoproteins in *M. bovis* remain underexplored due to their low sequence similarity with those of other bacteria and the scarcity of genetic manipulation tools for *M. bovis*. In this study, the lipoprotein LppA was identified in all examined *M. bovis* strains. Utilizing immunoelectron microscopy and Western blotting, it was observed that LppA localizes to the surface membrane. Recombinant LppA demonstrated dose-dependent adherence to the membrane of embryonic bovine lung (EBL) cells, and this adhesion was inhibited by anti-LppA serum. In vitro binding assays confirmed LppA’s ability to associate with fibronectin, collagen IV, laminin, vitronectin, plasminogen, and tPA, thereby facilitating the conversion of plasminogen to plasmin. Moreover, LppA was found to bind and enhance the accumulation of Annexin A2 (ANXA2) on the cell membrane. Disrupting LppA in *M. bovis* significantly diminished the bacterium’s capacity to adhere to EBL cells, underscoring LppA’s function as a bacterial adhesin. In conclusion, LppA emerges as a novel adhesion protein that interacts with multiple host extracellular matrix proteins and ANXA2, playing a crucial role in *M. bovis*’s adherence to host cells and dissemination. These insights substantially deepen our comprehension of the molecular pathogenesis of *M. bovis*.

**Supplementary Information:**

The online version contains supplementary material available at 10.1186/s13567-023-01242-1.

## Introduction

*Mycoplasma bovis*, a significant pathogen linked with bovine respiratory disease syndrome, instigates intense inflammatory responses, manifesting as pneumonia, mastitis, arthritis, otitis, and keratoconjunctivitis. Globally widespread, *M. bovis* inflicts considerable economic losses on the cattle industry [1]. *M. bovis* infection is implicated in various chronic diseases in feedlots, representing a formidable obstacle in the health management protocols of farms [2].

The adhesion to and invasion of host cells are crucial phases in the *M. bovis* infection process. *M. bovis* adheres to bovine tracheobronchial epithelial cells, which aids colonization of the bacteria within the host’s lungs [2]. Moreover, this pathogen can adhere to and penetrate diverse bovine cell types, potentially leading to an array of clinical diseases in cattle [3–5]. *M. bovis* engages with the extracellular matrix (ECM) and plasminogen, enhancing its adhesion, invasion, and spread within the host [6]. Nonetheless, the exact *M. bovis* factors integral to these interactions are yet to be distinctly understood. Variable surface protein (Vsp) has been recognized as a crucial adhesion-associated gene in *M. bovis*, known for provoking a robust immune reaction in the host [7, 8]. However, ongoing alterations in the expression, structure, and antigenicity of the Vsp protein result in an ineffective immune response, allowing *M. bovis* to consistently dodge the host’s antibody defenses [9, 10]. Although numerous *M. bovis* adhesion-related proteins, including NOX, MbfN, FBA, TrmFO, α-Enolase, MilA, P27, VpmaX, P26, and Mbov-0503, have been uncovered in recent times [11–21], devising an efficacious *M. bovis* vaccine remains problematic. Consequently, an immediate demand exists to pinpoint and investigate other potential candidate targets for vaccine development.

Membrane-associated proteins, especially lipoproteins, play a pivotal role in host–bacteria interactions [16, 22]. Presumed to be exposed to the extracellular environment, these proteins potentially facilitate not only the adhesion and invasion of pathogens into host cells but also the elicitation of immune responses [23]. Lipoproteins serve as key functional proteins for *Mycoplasma*, integral to various life processes of the microorganism, and stand as promising candidates for vaccine development [16]. Studies have identified lipoprotein LppA as a principal T-cell antigen in *Mycoplasma mycoides* subsp. *mycoides* [24]. An adenoviral vector expressing lipoprotein A (Ad5-LppA) elicited robust specific humoral and cell-mediated immune responses in mice, suggesting its potential as a vaccine against the severe respiratory disease contagious bovine pleuropneumonia [25]. However, the role of LppA in *M. bovis* adhesion and invasion remains unclarified. Most pathogenic microorganisms target ECM components from the host for adhesion and colonization [16, 26]. *Mycoplasma* can bind to ECM components such as collagen, laminin, fibronectin, plasminogen, and the glycosaminoglycan heparin [17, 27–29]. This interaction facilitates the adherence of pathogenic bacteria to host cells and fosters their subsequent invasion. *Mycoplasma* spp. is one of the smallest self-replicating organisms with reduced genomes. The lack of efficient genetic manipulation tools for these distinct organisms has impeded the characterization of virulence-related genes and the understanding of pathogenic mechanisms in these bacteria.

The present study investigated the role of LppA in *M. bovis* infection, thereby augmenting our comprehension of the pathogenic mechanisms at play in *M. bovis*. The study findings unveiled that LppA engages with host ECM components, enhances plasminogen activation through tissue plasminogen activator (tPA), and prompts the enrichment of ANXA2 protein on the cell membrane. These collective actions regulate the adhesion of *M. bovis* to host cells.

## Materials and methods

### Cells and strains

Embryonic bovine lung (EBL) cells and the HEK-293T cell line (commonly referred to as 293T) were cultured in Dulbecco’s Modified Eagle’s Medium (Gibco, USA), enriched with 10% fetal bovine serum (Gibco), and maintained at 37 °C in a 5% CO_2_ atmosphere. EBL cells, derived from the lung tissue of fetal cattle at approximately seven-months of gestation, serve as a significant cellular model for investigating viral and bacterial diseases in cattle [30]. The 293T cell line originates from HEK 293 cells and stably expresses the SV40 large T antigen [31]; it is frequently utilized for protein expression and the examination of protein–protein interactions in vitro. *M. bovis* was propagated in a pleuropneumonia-like organism (PPLO) medium supplemented with 20% horse serum (Gibco), following established protocols [32]. Cultivation of mutant strains required an additional 100 μg/mL of kanamycin, while the complemented strain, produced by transforming the ΔLppA mutant with the pIRR45-LppA plasmid, necessitated 5 μg/mL of puromycin. *Escherichia coli* DH5α and BL21(DE3) strains were employed for gene cloning and protein expression, respectively, with *E. coli* BL21 (DE3) recognized as a prevalent T7 expression host suitable for transformation and protein synthesis.

### Antibodies and reagents

Reagents such as mouse anti-fibronectin, donkey anti-mouse Alexa Fluor 488, and donkey anti-rabbit Alexa Fluor 647 were sourced from Thermo Fisher Scientific, USA. Rabbit anti-plasminogen was procured from AssayPro, USA. Rabbit antibodies against collagen IV, laminin, tPA, and vitronectin were supplied by Abcam, USA. Rabbit anti-HA, anti-ANXA2, and mouse anti-Flag antibodies were obtained from Proteintech Group, Inc., China. Biochemicals including bovine-derived fibronectin and plasminogen, as well as human-derived tPA, were purchased from Thermo Fisher Scientific, Cell Sciences, and Solarbio Science & Technology Co., Ltd., China, respectively. Human-derived collagen IV and laminin were acquired from Sigma-Aldrich, USA.

### DNA constructs and transfection

The complete sequence of *M. bovis LppA* (GenBank accession no. CP002188.1, MBOVPG45_0682) was optimized for *E. coli*-preferred codons (see Supplementary material) and synthesized by Sangon Biotech. Plasmids pIRR45-LppA, pCAGGS-FLAG-N-LppA, pCAGGS-HA-N-ANXA2, and pET30a-LppA were constructed using an In-Fusion kit (TaKaRa). Specifically, to complement the mutant, we developed a complementation plasmid, pIRR45, employing primer pairs pIRR45-TfibZT-F/R and pIRR45-LppA-F/R (Additional file 1) to amplify the *LppA* gene (inclusive of its presumed promoter) and subcloned it into the pIRR45 plasmid. The resulting pIRR45-LppA recombinant plasmid was introduced into the ΔLppA mutant strain via PEG 8000, generating the complemented Δ*LppA*:*LppA* strain. Plasmids pCAGGS-FLAG-LppA were created using primer pairs pCAGGS-FLAG-ZT-F/R and pCAGGS-FLAG-LppA-F/R (Additional file 1). The cattle ANXA2 gene was cloned into pCAGGS-HA using primer pairs pCAGGS-FLAG-ZT-F/R and pCAGGS-HA-ANXA2-F/R (Additional file 1), yielding pCAGGS-HA-ANXA2 plasmids. Both pCAGGS-FLAG-LppA and pCAGGS-HA-ANXA2 plasmids were co-transfected into 293T cells using Lipofectamine 3000 (Invitrogen, USA), following the manufacturer’s guidelines. The coding region of the *M. bovis LppA* gene underwent double digestion with *EcoR*I and *Xho*I restriction enzymes and was then ligated into the similarly digested pET30a-LppA plasmid. The derivative plasmid, pET30a-LppA, was introduced into *E. coli* strain BL21(DE3) through heat shock.

### Preparation of polyclonal antibodies against LppA

Polyclonal antibodies against *M. bovis* LppA were produced by immunizing 6-week-old Kunming mice with rLppA protein. Purified rLppA was emulsified with complete and incomplete Freund’s adjuvant (1:1, v/v), and the mice were immunized subcutaneously on four occasions at two-week intervals. Antisera were collected 2 weeks after the fourth immunization and titrated via enzyme-linked immunosorbent assay (ELISA). In brief, 96-well plates were coated with 10 μg/mL rLppA protein overnight at 4 °C. Following a block with 5% bovine serum albumin (BSA), double serial dilutions of both anti-LppA serum and pre-immune serum were applied to the wells and incubated at 37 °C for 30 min, succeeded by the addition of 100 µL of goat anti-mouse IgG-horseradish peroxidase (HRP) at a 1:10 000 dilution. After a 1-h incubation at room temperature, 3,3′,5,5′-tetramethylbenzidine (TMB) was added, and the reaction was terminated with H_2_SO_4_. Optical density (OD) readings were taken at 450 nm using an iMark microplate reader (Bio-Rad). The highest antibody dilution that met the criteria (OD value > 0.4) was recorded as the antibody titer. The specificity of the resulting polyclonal antibodies was confirmed through western blot analysis.

### Bioinformatics analysis

The NCBI database (Accessed on June 13, 2023) was utilized to search for *M. bovis* isolates from locations worldwide, yielding 262 isolates (Additional file 2). A comprehensive set of 150 572 proteome sequences was compiled into a self-constructed *M. bovis* protein database (MB-DATABASE). Similarly, the NCBI database (Accessed on June 13, 2023) was explored for *Mycoplasma* isolates from global locations, uncovering 89 distinct *Mycoplasma* isolates (Additional file 2). In total, 71 084 proteome sequences were assembled into a *Mycoplasma* self-constructed protein database (MY-DATABASE). An LppA Hidden Markov Model (LppA-HMM) was utilized through the hmmbuild function of the HMMER 3.0 software [33]. Upon retrieving the MB-DATABASE or MY-DATABASE using LppA-HMM, the screening criteria were established at an e-value < 10^−5^ to identify sequences similar to LppA. NCBI BLAST (blastp program) searches were executed with the acquired amino acid sequences. Amino acid sequences labeled as “LppA protein” and “Mycoplas_LppA” in NCBI annotation or domain annotation were chosen. Subsequent multiple sequence alignments were conducted employing MAFFT software [34], and a neighbor-joining phylogenetic tree was reconstituted with 1000 bootstraps using MEGA 11 software [35]. The LppA domain structure was examined using the SMART databases [36], and the structural characteristic model of LppA was formulated in AlphaFold v2.0 [37]. The 1–350 amino acid sequence of LppA was scrutinized for antigenic epitope regions using Protean software (Lasergene; DNAStar 6.0).

### *Mycoplasma* adhesion assay

EBL cells were allocated into a 12-well plate (1 × 10^5^ cells per well) and incubated in 5% CO_2_ at 37 °C overnight. *M. bovis* was introduced to the EBL cells at a multiplicity of infection of 1000 and incubated for 1.5 h, after which the cells were rinsed thrice with 1 × phosphate-buffered saline. The cells were subsequently digested with TrypLE™ Express (Gibco), and dilutions were distributed on PPLO agar plates. Viable *Mycoplasma* cells were quantified by counting colonies following 3–5 days of cultivation.

### Confocal microscopy and immunoelectron microscopy

EBL/293T cells were stabilized with 2% paraformaldehyde, permeabilized with 0.1% Triton X-100, and subsequently incubated with the specified antibodies. Images were captured using a Leica laser scanning confocal microscope and processed with LAS-AF-Lite 3.3.0 software. Immunogold electron microscopy was employed to delineate the subcellular localization of LppA. In brief, washed *M. bovis* cells were secured in 2% paraformaldehyde and 0.5% glutaraldehyde and processed at the Lilai Biomedicine Experiment Center (Chengdu, China). For immunogold labeling, mouse anti-LppA serum served as the primary antibody, while goat anti-mouse IgG linked to 10-nm colloidal gold particles (Beijing Biosynthesis Biotechnology) was utilized as the secondary antibody. The ultimately desiccated samples were examined using transmission electron microscopy (JEM-1400FLASH).

### Immunoprecipitation (IP)

#### IP-mass spectrometry (IP-MS)

rLppA protein (10 μg) was incubated with EBL whole-cell lysates overnight at 4 °C. Anti-LppA serum was introduced to the mixture and rotated at 4 °C for 8 h. Immunoprecipitation experiments were conducted using protein A/G magnetic beads following the manufacturer’s instructions (Invitrogen), and the results were analyzed by sodium dodecyl sulfate–polyacrylamide gel electrophoresis (SDS-PAGE) with silver staining and mass spectrometry. Briefly, 25 µL (0.25 mg) of Pierce Protein A/G Magnetic Beads were placed in a 1.5 mL microcentrifuge tube. 175 µL of 1 × TBS containing 0.05% Tween-20 (TBST) was added, and the tube was gently shaken for mixing. The tube was then placed in a magnetic stand to gather the beads, and the supernatant was discarded following a 1-min wash with 1 mL of TBST. The beads were collected with a magnetic stand, and the supernatant was removed. The mixture of Anti-LppA serum and rLppA protein was added to the tube containing pre-washed magnetic beads and incubated at room temperature for 1 h with agitation. The beads were subsequently immobilized using a magnetic stand, and the supernatant was reserved for analysis. The magnetic beads underwent two washes with 500 µL of TBST, followed by two washes with 500 µL of purified water. After each wash, the beads were collected using a magnetic stand, and the supernatant was discarded. After removal from the magnetic stand, 100 µL of 1 × SDS-PAGE loading buffer was added to the tube, thoroughly mixed, and heated at 95 °C for 10 min. Finally, the supernatant containing the target antigen was magnetically separated from the beads, and the supernatant was retained. Silver staining was executed using the Fast Silver Stain kit (Beyotime) per the manufacturer’s guidelines. Mass spectrometry analysis was conducted by Bioprofile (Shanghai) Technology Co., Ltd.

#### Co-IP

To investigate the LppA protein’s interaction with ANXA2, 293T cells were seeded into 6-well culture plates and transfected with pCAGGS-FLAG-N-LppA and pCAGGS-HA-N-ANXA2 plasmids. Transfected cells were harvested at 24 h post-transfection and lysed in NP-40 Lysis Buffer containing 1 mM protease inhibitor. The lysates were centrifuged at 12 000 × *g* for 10 min, and the supernatants, containing 500 μg of total protein, were incubated overnight with 10 μg of mouse monoclonal antibody against Flag, with gentle agitation at 4 °C. Immunoprecipitation experiments were conducted with Protein A/G magnetic beads as previously detailed. Western blot detection was carried out using anti-HA rabbit polyclonal antibody.

### Western blot and dot blot analyses

#### Western blot

Protein samples were separated on a 10% SDS-PAGE and transferred to polyvinylidene fluoride membranes (Amersham). The membranes were blocked with 5% BSA for 1 hat room temperature, followed by overnight incubation with primary antibodies at 4 °C. After three washes with TBST), the membranes were exposed to HRP-coupled goat anti-mouse antibody (Abcam, 1:5000 dilution) or HRP-coupled goat anti-rabbit antibody (Abcam, 1:5000 dilution) for 1 h at room temperature. Detection was performed using ECL (Thermo Fisher Scientific), and the signal was captured with the ChemiDoc imaging system (Bio-Rad).

#### Dot blot

Serial twofold dilutions of rLppA protein and 6 × His peptides (5.0–0.3125 μg) were applied onto nitrocellulose (NC) membranes (Solarbio, China). After drying at room temperature for 1 h, the NC membranes were blocked with 5% BSA for 1 h at room temperature. They were then incubated with 10 μg of fibronectin, collagen IV, laminin, vitronectin, tPA, or plasminogen, respectively, on a rocking platform overnight at 4 °C. The membranes were washed three times for 10 min each with 1 × TBST. Primary antibodies such as anti-fibronectin, anti-collagen IV, anti-laminin, anti-vitronectin, anti-tPA, or anti-plasminogen IgG were added and incubated for 1 h at room temperature. Subsequently, Goat anti-mouse HRP-IgG or goat anti-rabbit HRP-IgG was applied as secondary antibodies for 1 h at room temperature, followed by ECL detection. Signals were captured using the ChemiDoc imaging system.

### Enzyme-linked immunosorbent assay

ECM components (fibronectin, collagen IV, laminin, and vitronectin; 300 ng/well) or EBL cell membrane extracts (200 ng per well) were coated onto a 96-well plate and incubated at 4 ℃ overnight. For the cell membrane microplate binding assay, various dilutions (200–0.8 ng) of rLppA protein were introduced into ELISA wells previously coated with EBL cell membrane extracts. For the ECM microplate binding assay, rLppA protein was serially diluted (250–1.95 ng) and applied to ELISA wells coated with ECM proteins. In the adhesion inhibition test, different dilutions of anti-LppA mouse serum (1:10–1:1280 dilution) were initially combined with *M. bovis* PG45 and incubated at 37 °C for 1 h; thereafter, the mixture was introduced to ELISA wells coated with EBL cell membrane extracts. Anti-LppA serum and goat anti-mouse HRP-IgG served as primary and secondary antibodies, respectively. The chromogenic substrate used was TMB, and the OD was measured at 405 nm using an iMark microplate reader. BSA treatment served as a negative control.

### Plasminogen activity assay

The rLppA protein (1 μg) was incubated with plasminogen (1 μg) at 37 °C for 1 h, followed by its addition to a 96-well plate. Subsequently, tPA (50 ng) or the lysine analog aminoacetic acid ε-ACA (80 mM) was introduced and the mixture was incubated for 15 min at 37 ℃. Absorbance changes at 405 nm were monitored at 15-min intervals using an iMark microplate reader, following the addition of 0.5 mM of the specific substrate D-Val-Leu-Lys *p*-nitroaniline dihydrochloride.

### ANXA2 translocation assay

The pCAGGS-FLAG-N and pCAGGS-FLAG-N-LppA plasmids were transfected into EBL cells for 24 h, respectively. The membrane and cytoplasm of EBL cells, either transfected with plasmid or infected with *M. bovis*, were separately extracted using a commercial Membrane Protein Extraction Kit (Thermo Fisher Scientific). Changes in the expression of ANXA2 in the cell membrane and cytoplasm were detected using western blot using an anti-ANXA2 antibody. α-Tubulin and Na^+^/K^+^-ATPase were employed as marker proteins for the cytoplasm and membrane, respectively.

### Statistical analysis

Each experiment was independently replicated three times, and each sample was analyzed in triplicate. Statistical analyses were conducted using unpaired *t*-tests and one-way/two-way analysis of variance with GraphPad Prism 6.0 software. Significant differences were indicated as follows: *p < 0.05, **p < 0.01, ***p < 0.001, and ****p < 0.0001. The term “ns” denotes a lack of statistical significance.

## Results

### Sequence and immunogenicity analyses of LppA

**Figure 1:**
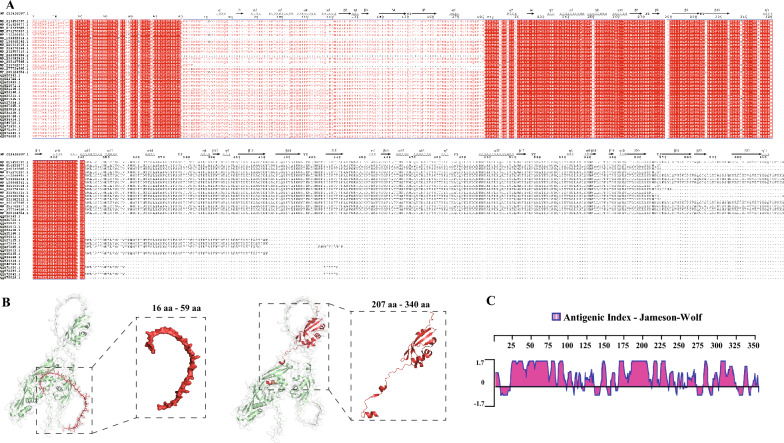
**Sequence characterization of LppA in** ***M. bovis.*** **A** Multiple conserved sites of LppA in *M. bovis*. Highly conserved residues are shown in red. **B** Protein structures of LppA were modeled using AlphaFold2; red indicates the most conserved region. **C** Multiple epitopes of 1–340 amino acids of *M. bovis* LppA conserved regions predicted using DNAstar 6.0 software.

Genome analysis has revealed that the LppA gene is universally present in *M. bovis* isolates, establishing it as a core gene of this species. *M. bovis* LppA exhibits highly conserved amino acid sequences within the 1–340 amino acid (aa) region, particularly in the 16–59 aa and 207–340 aa regions, which are completely consistent across all *M. bovis* strains (Figure 1A). These regions may represent the core functional areas of *M. bovis*’s LppA. Throughout its evolution, LppA from different *M. bovis* isolates has experienced deletions in various regions, most notably, significant losses of amino acid segments within the 65–202 aa or 341–617 aa regions (Figure 1A). Phylogenetic analysis, based on LppA’s amino acid sequence in *Mycoplasma*, demonstrated that LppA divides into five main groups, with *M. bovis* and *Mycoplasma agalactiae* clustering closely together (Additional file 3A). Further multiple sequence alignment analysis revealed that LppA is highly conserved among *Mycoplasma* species, with numerous conserved sites suggesting similar functions across different *Mycoplasma* species (Additional file 3B). Functional domain prediction analysis of LppA, using SMART software, identified a signal peptide at the N-terminal 1 to 21 aa. Analysis of LppA’s tertiary structure with AlphaFold 2 indicated that the 207–340 aa region contains multiple helical structures (Figure 1B).

To determine whether LppA serves as a potential antigenic target for *M. bovis*, we identified several antigenic sites within LppA’s highly conserved amino acid region through bioinformatics analysis (Figures 1A and C). Anti-LppA mouse serum was produced following procedures for prokaryotic expression, recombinant protein induction, and polyclonal antibody generation (Additional file 4). We observed that rLppA reacted with bovine serum from natural and experimental *M. bovis* infections, as well as with anti-LppA mouse serum, but not with *M. bovis*-negative bovine serum (Additional file 5A). LppA from *M. bovis* was successfully detected in all six clinical isolates tested (Additional file 5B). These results suggest that LppA is highly immunogenic and could serve as a vaccine candidate for *M. bovis*.

### Surface localization of *M. bovis* LppA

**Figure 2:**
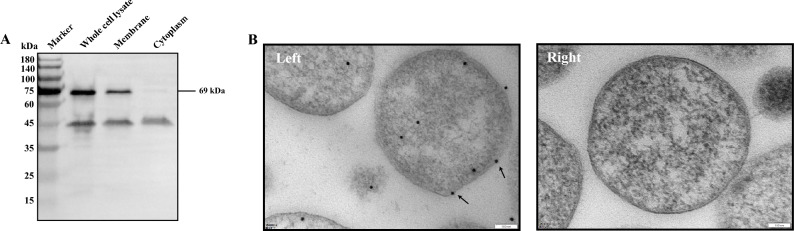
**Surface localization of LppA in** ***M. bovis.*** **A** Distribution of LppA in *M. bovis* by immunoblotting assay. The 180-kDa protein marker (M) is labeled. Total protein (lane 1), cell membrane fraction (lane 2), and cytoplasmic fraction (lane 3) of *M. bovis* were subjected to western blotting with anti-LppA serum. **B** Immunoelectron microscopy localization of LppA in *M. bovis*. (Left) Anti-LppA mouse serum was used as the primary antibody. Black arrows indicate colloidal gold particles. (Right) Negative control using pre-immune mouse serum. Scale bar for transmission electron microscopy image: × 150 000 magnification, 100 nm.

To ascertain LppA’s localization, we separately extracted the membrane proteins and cytoplasmic proteins of *M. bovis* (Figure 2A). LppA was primarily located in the cell membrane of *M. bovis*, with lesser quantities found in the cytoplasm. This membrane-centric localization of LppA was further confirmed through an immunogold labeling assay (Figure 2B).

### LppA as a novel adhesin in *M. bovis*

**Figure 3:**
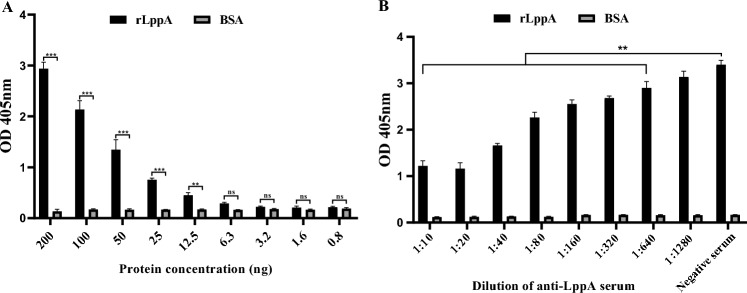
**Binding of** ***M. bovis *LppA to EBL cell membrane extracts. A** rLppA binds to embryonic bovine lung (EBL) cell membrane extracts in a dose-dependent manner. Different concentrations of rLppA (200–0.8 ng) were added to the wells of plates coated with EBL cell membrane extracts (200 ng) and were then incubated with anti-LppA serum for 1 h, followed by incubation with horseradish peroxidase–conjugated goat anti-mouse secondary antibody. Subsequently, after 10 min of incubation with tetramethylbenzidine, bound rLppA was detected by measuring the optical density of the wells at 405 nm using a microtiter plate reader. Bovine serum albumin served as the negative control. **B** Inhibition of rLppA binding to EBL cell membranes by anti-LppA serum. rLppA (200 ng) was pre-incubated with various concentrations of anti-LppA serum for 1 h and added to wells coated with EBL cell membrane extracts (200 ng). Pre-immune serum was used as the negative control. Values are presented as mean ± standard error of the mean of three independent experiments (*p < 0.05, **p < 0.01, ***p < 0.001).

The interaction between rLppA and membrane extracts from EBL cells was analyzed using ELISA. rLppA was found to bind to the EBL cell membrane in a dose-dependent manner within the range of 12.5–200 ng, compared with the control group (Figure 3A). This binding was inhibited by anti-LppA serum at a dilution of 1:640 (*p* < 0.0001) (Figure 3B). These data indicate that LppA directly interacts with EBL cells, identifying it as a novel cytoadhesin of *M. bovis*.

### LppA binds host ECM components

**Figure 4:**
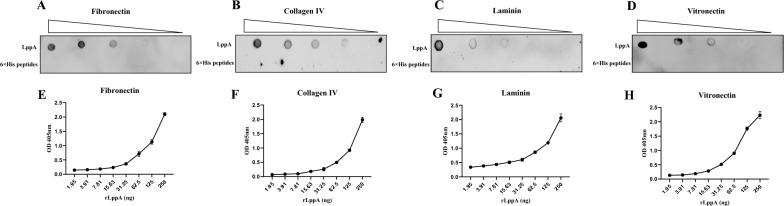
**Binding of** ***M. bovis*** **LppA to host ECM components. A**–**D** Binding ability of *M. bovis* LppA to host extracellular matrix components (fibronectin, collagen IV, laminin, and vitronectin) was tested by dot blot, with 6 × His peptides as the negative control. **E**–**H** LppA binds to fibronectin, collagen IV, laminin, and vitronectin in a dose-dependent manner. Different concentrations of rLppA (250–1.95 ng) were added to wells coated with fibronectin, collagen IV, laminin, or vitronectin (200 ng). The optical density was measured at 405 nm.

To further investigate LppA’s adhesion to host cells, we evaluated its potential interaction with ECM components. Both dot blot analysis and ELISA were utilized to assess rLppA’s binding affinity for fibronectin, collagen IV, laminin, and vitronectin, using 6 × His peptides as a negative control. The results indicated that rLppA could bind to fibronectin, collagen IV, laminin, and vitronectin in a dose-dependent manner (Figure 4).

### LppA promotes plasminogen activity by binding host plasminogen and tPA

**Figure 5:**
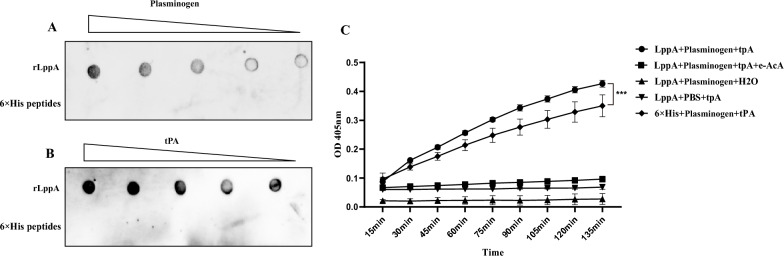
**Activation of plasminogen bound to LppA. A**, **B** Binding ability of *M. bovis* LppA to plasminogen and tPA was detected by dot blot, with 6 × His peptides acting as the negative control. **C** Determination of kinetic curves of LppA-promoted plasminogen activation by tPA. Plasminogen and rLppA were incubated on a microtiter plate for 1 h, and then tPA and the chromogenic substrate d-Val-Leu-Lys p-nitroaniline dihydrochloride were added. Plasmin activity was detected photometrically at 405 nm for every 15 min for 135 min. Wells without plasminogen or tPA were used as negative controls. rLppA-bound plasminogen was converted to plasmin, which cleaved the substrate d-Val-Leu-Lys p-nitroaniline dihydrochloride in a time-dependent manner. ε-ACA inhibited d-Val-Leu-Lys p-nitroaniline dihydrochloride cleavage. The absorbance at 405 nm of the 6 × His peptide control was significantly lower than that of the rLppA-added wells.

rLppA’s ability to bind plasminogen in a dose-dependent manner was demonstrated through a dot blot test (Figure 5A). The plasmin-specific chromogenic substrate d-Val-Leu-Lys 4-nitroanilide dihydrochloride was employed to detect the activation of plasminogen bound to rLppA by the activator tPA. Wells containing rLppA protein, plasminogen, and tPA exhibited higher OD values at 405 nm compared to control wells with 6 × His peptides (Figure 5C). This suggests that rLppA enhances the efficiency of plasminogen conversion to active plasmin. The trend of tPA-mediated plasminogen activation was observed both in the presence and absence of rLppA. The inclusion of rLppA significantly accelerated the rate of plasminogen activation from 15 to 120 min compared to wells containing only plasminogen and tPA (*p* < 0.0001; Figure 5C). However, no increase in OD values was noted in wells containing only plasminogen and rLppA or rLppA and tPA (Figure 5C), indicating that rLppA solely enhanced tPA’s activation of plasminogen and did not possess the functionalities of tPA or plasmin. The OD values in wells with rLppA, tPA, and plasminogen significantly decreased upon incubation with the lysine analog ε-ACA (Figure 5C), suggesting ε-ACA also affects rLppA’s activation capacity. Additionally, rLppA was observed to bind to tPA (Figure 5B).

### Annexin A2 is a cellular receptor of LppA

**Figure 6:**
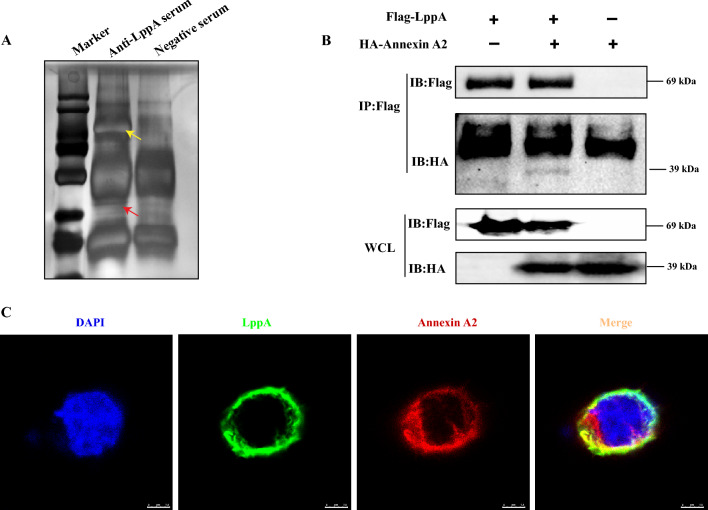
**Annexin A2 is a cellular receptor of** ***M. bovis *LppA. A** LppA-immunoprecipitated proteins identified by immunoprecipitation-mass spectrometry (IP-MS) assays. Yellow arrowheads indicate LppA; red arrowheads indicate host proteins that interact with LppA. **B** Co-immunoprecipitation analysis. Lysates from 293T cells (+) transfected with pCAGGS-FLAG-N-LppA and pCAGGS-HA-N-ANXA2 plasmids were immunoprecipitated using an anti-Flag antibody and analyzed using western blotting with antibodies to FLAG and HA. Lysates from 293T cells (−) not transfected with pCAGGS-FLAG-N-LppA or pCAGGS-HA-N-ANXA2 plasmids were used as the control group. Whole-cell lysates (WCL) of 293T were used as the input. **C** Co-localization of the LppA and ANXA2 proteins was analyzed using confocal microscopy. 293T cells were co-transfected with pCAGGS-FLAG-N-LppA and pCAGGS-HA-N-ANXA2 plasmids and labeled with mouse anti-HA and rabbit anti-Flag, followed by Alexa-Fluor-conjugated donkey anti-mouse (488) and donkey anti-rabbit (647) secondary antibodies. The cells were then observed using a confocal microscope. Green represents LppA, red represents ANXA2, and blue represents the nuclear stain 4′,6-diamidino-2-phenylindole (DAPI); co-localization assay suggests that LppA is almost completely coincident with ANXA2.

To discern additional host proteins interacting with *M. bovis* LppA, we employed IP-MS and identified several potential LppA-interacting proteins (Figure 6A). LppA’s interaction with ANXA2 was substantiated through Co-IP (Figure 6B), and immunofluorescence microscopy revealed co-localization of LppA and ANXA2 in 293T cells (Figure 6C). These findings confirm that LppA interacts with the ANXA2 protein.

### LppA promotes the enrichment of ANXA2 protein on the cell membrane

**Figure 7:**
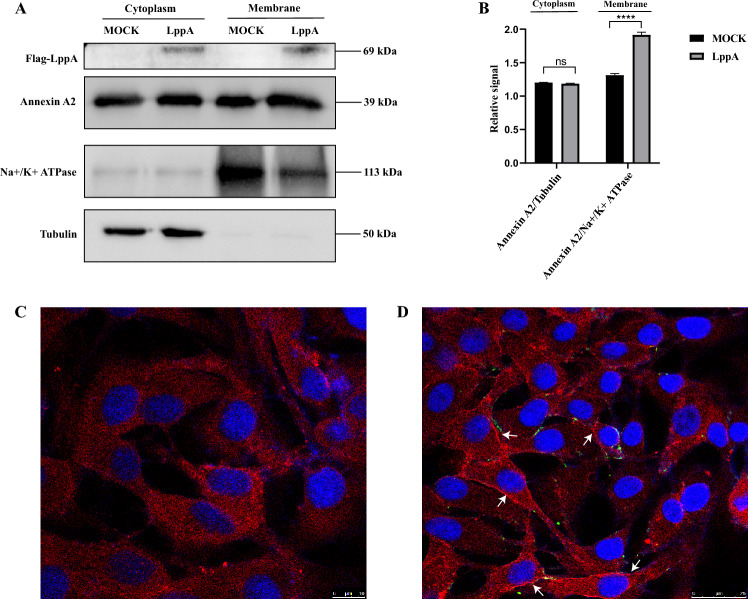
**LppA promotes the enrichment of ANXA2 on the cell membrane.** The pCAGGS-FLAG-N and pCAGGS-FLAG-N-LppA plasmids were separately transfected into embryonic bovine lung (EBL) cells for 24 h. **A** Expression changes of ANXA2 in the cell membrane and cytoplasm were detected using an anti-ANXA2 antibody. α-Tubulin and Na+/K+-ATPase were used as marker proteins for the cytoplasm and cell membrane, respectively. **B** Relative ANXA2 expression in the cell membrane and cytoplasm. **C** EBL cells transfected with the pCAGGS-FLAG-N plasmid. **D** EBL cells transfected with the pCAGGS-FLAG-N-LppA plasmid. Blue represents DAPI, red represents ANXA2, and green represents rLppA. The white arrow indicates the enrichment of the ANXA2 protein on the cell membrane.

The expression levels of ANXA2 in the cytoplasm and cell membrane of EBL cells were assessed through Western blot analysis, with α-tubulin and Na+/K+-ATPase serving as markers for cytoplasmic and membrane proteins, respectively. ANXA2 expression levels were similar in the cytoplasmic and membrane fractions of either uninfected EBL cells or cells transfected with the pCAGGS-FLAG-N plasmid (Figure 7A and Additional file 6A). However, in cells infected with *M. bovis* or transfected with the pCAGGS-FLAG-N-LppA plasmid, there was a notable enrichment of ANXA2 in the membrane, suggesting an increased presence of ANXA2 on the cell surface (Figure 7A and Additional file 6A). This observation was corroborated by confocal microscopy, which showed a substantial increase in the ANXA2 fluorescence signal on the surface of cells either transfected with the pCAGGS-FLAG-N-LppA plasmid or infected with *M. bovis* PG45, compared to control cells transfected with the pCAGGS-FLAG-N plasmid or uninfected cells (Figures 7B, C, D and Additional files 6B, C, D). These findings imply that LppA might assist *M. bovis* in promoting the accumulation of ANXA2 on the cell membrane.

### Disruption of the LppA gene markedly reduces the adhesion of *M. bovis* to cells in vitro

**Figure 8:**
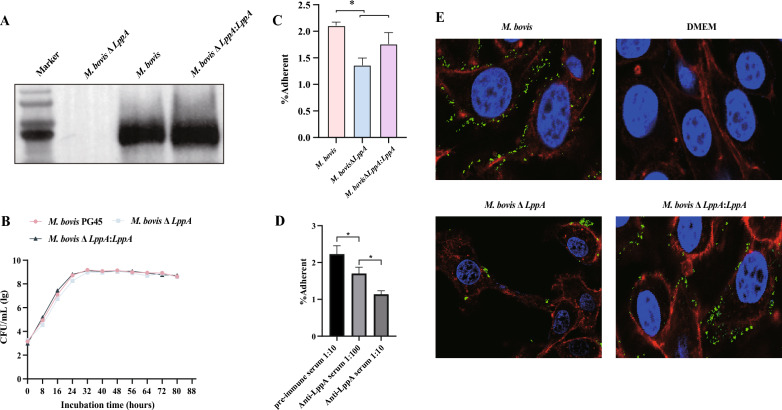
**LppA is a novel adhesion protein of* M. bovis.*** **A** Presence of LppA in *M. bovis* wild-type, *M. bovis* Δ*LppA*, and *M. bovis* Δ*LppA:LppA* was examined using anti-LppA mouse serum. **B** Comparative growth curves of *M. bovis* wild-type, *M. bovis* Δ*LppA*, and *M. bovis* Δ*LppA*:*LppA*. **C** *M. bovis* wild-type, *M. bovis* Δ*LppA*, and *M. bovis* Δ*LppA*:*LppA* were used to infect embryonic bovine lung (EBL) cells for 1.5 h. Subsequently, the cells were washed three times with phosphate-buffered saline, digested with trypsin, and the adherent mycoplasmas were counted. **D** Adhesion inhibition assay of anti-LppA serum to *M. bovis*. *M. bovis* was pre-incubated with various concentrations of anti-LppA serum for 1 h and then used to infect EBL cells. Pre-immune serum was used as the control. Values are presented as mean ± standard error of the mean of three independent experiments (*p < 0.05, **p < 0.01, ***p < 0.001). **E** *M. bovis* wild-type, *ΔLppA*, and *ΔLppA:LppA* were stained with CFDA-SE for 30 min and then used to infect EBL cells. The nuclei of the cells were stained with DAPI and the cytoskeleton was stained with rhodamine phalloidin. Green represents *M. bovis*, red represents the cytoskeleton, and blue (DAPI) represents the nucleus.

The LppA-deficient *M. bovis* mutant (Δ*LppA*) was generated using the transposon mutagenesis method (Additional file 7). The Δ*LppA*:*LppA* complementation strain was subsequently created by transforming *ΔLppA* mutants with the pIRR45-LppA plasmid. Polymerase chain reaction and Sanger sequencing, utilizing specific primers (Additional file 1), confirmed the insertion of the transposon into 47.9% of the full coding sequence of the LppA gene downstream of the start codon in the Δ*LppA* mutants (spanning nucleotides 880–881). The expression levels of LppA in the Δ*LppA* and Δ*LppA*:*LppA* strains were validated through Western blot analysis using anti-LppA serum (Figure 8A). Growth curves for *M. bovis* wild-type, Δ*LppA*, and Δ*LppA*:*LppA* strains revealed no significant differences, suggesting that LppA’s absence does not impede *M. bovis* growth (Figure 8B).

The adhesion capability of *M. bovis* wild-type, Δ*LppA*, and Δ*LppA*:*LppA* strains to EBL cells was then examined. The Δ*LppA* mutant displayed a significantly diminished adhesion to EBL cells compared with the parental strain PG45, but this adhesion trait was partially reinstated in the complemented Δ*LppA*:*LppA* strain, as further evidenced by confocal microscopy analysis (Figure 8C, E). Pre-incubating *M. bovis* with anti-LppA serum before infecting EBL cells also markedly reduced *M. bovis*’s adhesion to EBL cells (Figure 8D). This adhesion inhibition was dose-dependent, with a 1/10 dilution of anti-LppA serum resulting in more pronounced inhibition compared to a 1/100 dilution (*p* < 0.0001). Additionally, a 1/100 dilution of anti-LppA serum significantly curtailed *M. bovis*’s adhesion capacity compared to pre-immune mouse serum (*p* < 0.0001). These outcomes highlight LppA’s crucial role in facilitating the adhesion of *M. bovis* to EBL cells.

## Discussion

Adhesion to and colonization of host cells are crucial biological processes for *Mycoplasma* to infect the host. However, the specific virulence factors utilized by *M. bovis* for adhesion, invasion, and immune evasion remain unidentified. A significant obstacle impeding the functional annotation of *Mycoplasma* genes is their low sequence similarity to those of other bacteria, compounded by the scarcity of effective genetic manipulation tools for *Mycoplasma* [1]. Several proteins, including enolase, TrmFo, Mbov_0503, and mbfN, have been previously identified as adhesion-related proteins in *M. bovis* [11–21]. Nevertheless, only a few proteins have been validated as cytoadhesion-related proteins at the bacterial level. Consequently, research into the molecular mechanisms of *Mycoplasma* pathogenicity trails significantly behind that of other pathogenic bacteria. In this study, we evaluated the adhesion of *M. bovis* PG45, Δ*LppA*, and Δ*LppA*:*LppA* to EBL cells. The adhesion of Δ*LppA* to EBL cells was notably reduced compared to that of the parental strain and Δ*LppA*:*LppA*. Treating *M. bovis* PG45 with a specific anti-LppA serum led to a substantial decrease in adhesion to EBL cells. Together, these findings suggest that LppA is a novel adhesion-related protein in *M. bovis*.

Surface-localized adhesion-associated proteins in *M. bovis* present potential targets for developing drugs, vaccines, and immunodiagnostic assays against this economically significant ruminant pathogen. In this research, immunoelectron microscopy and Western blot analysis demonstrated that LppA is primarily located on the cell membrane surface of *M. bovis*. LppA is immunogenic and recognized by the host to trigger an immune response. Recent studies have shown that the LppA of *Mycoplasma mycoides*, as a primary antigen, can provoke a robust immune reaction in the host, such as a T-cell response, during the initial infection stages [24, 38]. In other pathogenic bacteria such as *Salmonella enterica* serovar Typhimurium and *Mycoplasma mycoides* subspecies *mycoides*, LppA also serves as a powerful inducer of host inflammatory and immune responses [25, 39]. In recent years, several immunogenic surface adhesion proteins of *M. bovis*, such as Vsp [8, 21], have been uncovered. However, the expression of the Vsp family of proteins is variable. In different *M. bovis* isolates, the expression patterns of the Vsp family differ, contributing significantly to the immune evasion tactics of *M. bovis* [40]. LppA-related proteins are commonly found in *M. bovis* strains, and the 1–340 amino acid region is highly conserved, containing numerous antigenic epitopes. Hence, the newly characterized adhesion protein LppA of *M. bovis* highlighted in this study emerges as a potential candidate target for drugs, vaccines, and immunodiagnostic development. Future work will explore the role of LppA in the pathogenesis of *M. bovis* infections in cattle.

Phylogenetic and sequence analyses showed that LppA-related proteins presented five main groups in *Mycoplasma*, among which *M. bovis* and *Mycoplasma agalactiae* clustered together. Multiple sequence alignment analysis has shown that LppA possesses numerous identical conserved sites across *Mycoplasma* species, and these sites maintain similar spatial conformations in the protein’s tertiary structure. Therefore, these domains might be critical to LppA’s biological function. Interestingly, some *M. bovis* strains exhibit a partial absence of the amino acid sequence (amino acids 65–202 or 341–617) at the carboxy-terminus of LppA. LppA is not unique in this aspect; similar fragment deletions have been observed in MbfN, a leucine-rich repeat lipoprotein of *M. bovis* [16]. The absence of these protein fragments might induce functional alterations in *M. bovis*. Nevertheless, the reasons for these deletions and the functions of the missing amino acid sequences remain to be explored. Genetic closeness between *M. bovis*/*Mycoplasma agalactiae* and the *Mycoplasma mycoides* cluster was noted in phylogenetic and sequence analyses, suggesting the possibility of lateral gene transfer among them. The presence of LppA-like sequences in other bacteria and their evolutionary relationships warrant further investigation.

The host ECM is central to numerous biological processes, including cell adhesion, differentiation, growth, and migration. However, components of the host ECM also serve as common facilitators in pathogen–host cell interactions [41, 42]. Pathogens initiate contact with a host through the interaction of their virulence factors and ECM components, a critical step in the infection process [16]. *Leptospira* is known to bind to ECM components such as collagen, fibronectin, and laminin [43, 44], with certain bacterial proteins identified as mediating these interactions [44]. For instance, the LigA and LigB proteins of *Leptospira interrogans* serovar Copenhageni facilitate the bacterium’s binding to fibronectin, laminin, type IV collagen, and the plasma protein fibrinogen [43]. Similarly, the ability of *S. aureus* to bind to fibronectin constitutes a significant virulence trait, indicative of the bacterium’s invasive capacity [45]. Previous research indicated that *M. bovis* secures and perpetuates its infection in host cells by adhering to multiple ECM receptors [6, 16]. In this study, we discovered that rLppA binds to fibronectin, collagen IV, laminin, and vitronectin in a dose-dependent fashion, underscoring LppA’s pivotal role in *M. bovis* adhesion to host cells through interactions with ECM components.

Plasminogen and ECM proteins share a close relationship within the host [46]. Plasminogen, a vital source of plasmin, is central to the fibrinolytic system. This extensive proteolytic system plays a crucial role in various physiological processes, such as the breakdown of fibrin clots and the degradation of various ECM and connective tissue components [47]. Numerous pathogenic microorganisms express factors that can bind to and enhance plasminogen activity [48–50], allowing pathogens to co-opt the host’s fibrinolytic system to aid their invasion and colonization [47, 49, 51]. For instance, GAPDH and enolase in *Mycoplasma hyorhinis* serve as plasminogen receptors (PlgRs), binding and activating plasminogen to assist the pathogen in degrading the host’s ECM [27–29]. This interaction may be a potential mechanism through which *M. hyorhinis* breaches tissue barriers in vivo, leading to serous fibrinous inflammation in piglets’ body cavities and joints [29]. This study investigated the role of *M. bovis*’s LppA in plasminogen hijacking and identified an interaction between plasminogen and rLppA. rLppA-bound plasminogen, once activated by tPA, forms plasmin, degrading specific substrates. The lysine analog ε-ACA impeded the activation of LppA-bound plasminogen, indicating that lysine residues are vital functional sites at which LppA promotes the conversion of plasminogen to plasmin. These findings suggest LppA is a novel PlgR in *M. bovis*, potentially aiding the pathogen’s tissue dissemination.

ANXA2, a member of the extensive annexin family of Ca^2+^-regulatory and phospholipid-binding proteins, participates in several cellular physiological processes, such as membrane dynamics, cell–cell interactions, and cell adhesion [52, 53]. Although primarily found in the cytoplasm, ANXA2 can traverse the cell membrane to perform various functions, including enhancing plasmin activities [52–55]. Several studies have documented microorganisms’ interactions with ANXA2. It has been identified as a novel receptor for the adhesion of spotted fever group *Rickettsia* and *Staphylococcus aureus* to vascular endothelial cells [56] and is known to interact with the CARDS toxin of *Mycoplasma pneumoniae*, promoting host cell vacuolization [57], and heat shock protein GroEL of *Mycoplasma gallisepticum* interacts with ANXA2, facilitating host infection [58]. ANXA2 also engages with *M. hyorhinis* p37, activating the NF-κB pathway to mediate infection [59]. Recent research indicated that *M. bovis* can utilize ANXA2 for adhesion, invasion, and the downregulation of proteins essential for the IL-17 signaling pathway [60]. However, the specific *M. bovis* factors that interact with host ANXA2 were previously unidentified. In this study, we discovered an interaction between LppA and ANXA2 through IP-MS and Co-IP analyses, noting that LppA fosters the enrichment of ANXA2 on the EBL cell membrane. This suggests LppA assists *M. bovis* in adhering to the host through interactions with the host’s ECM and ANXA2.

In conclusion, this study identified LppA as a multifunctional surface protein on *M. bovis*, capable of adhering to host cells. LppA interacts with multiple ECM components, host plasminogen, and ANXA2, promoting *M. bovis* colonization and spread during infection. These insights enhance our understanding of *M. bovis* pathogenesis and offer new perspectives for preventing and controlling *M. bovis* infections.

### Supplementary Information

**Additional file 1. Plasmids, oligonucleotides, cell lines and strains used in this study.** List of plasmids, primers, *M. bovis* strains, and cell line used in this study.

**Additional file 2. The strains information of MB-DATABASE and MY-DATABASE.** NCBI accession numbers of strains used to construct the MB-DATABASE and MY-DATABASE databases. Genetic analysis of LppA sequences in *Mycoplasmas* and bacteria.

**Additional file 3. Sequence characterization of LppA in different species of the genus Mycoplasma.** **A**. Phylogenetic tree constructed based on LppA sequences was divided into five groups based on the topological structure. Groups 1 to 5 are marked with yellow, blue, purple, pink, and green, respectively. The LppA of *M. bovis* and *M. agalactiae* clustered in Group 1. **B.** Multiple conserved sites of LppA in *Mycoplasma* were revealed. Highly conserved residues are shown in red. **C.** Protein structures of *M. bovis* LppA were modeled using AlphaFold2; red indicates the most conserved region.

**Additional file 4. Recombinant LppA expression and polyclonal antibody preparation.** **A**. Purification of the rLppA protein washed with different concentrations of imidazole solution and analyzed using sodium dodecyl sulfate–polyacrylamide gel electrophoresis. **B.** LppA of *M. bovis* PG45 was examined by western blot using anti-LppA mouse serum and negative serum.

**Additional file 5. Immunogenicity analysis of LppA in** ***M. bovis.*** **A**. rLppA protein was examined using western blot using different sera. *M. bovis*-negative bovine serum was used as the negative control. **B.** Presence of LppA was examined in six strains of *M. bovis* by using anti-LppA serum.

**Additional file 6.** ***M. bovis *infection promotes ANXA2 translocation from the cytoplasm to the membrane.** The embryonic bovine lung (EBL) cells were infected with *M. bovis* PG45 for 24 h. **A.** Expression changes of ANXA2 in the cell membrane and cytoplasm were detected by using an anti-ANXA2 antibody. α-Tubulin and Na + /K + -ATPase were used as marker proteins for the cell cytoplasm and membrane, respectively. **B.** Relative ANXA2 expression in the cell membrane and cytoplasm. **C.** EBL cells uninfected with the *M. bovis* PG45. **D.** EBL cells infected with the *M. bovis* PG45. Blue represents DAPI, red represents ANXA2, and green represents *M. bovis*. The white arrow indicates ANXA2 translocation from the cytoplasm to the membrane.

**Additional file 7. The methods of construction and identification of *M. bovis *PG45 mutant strains, expression and purification of the recombinant protein, extraction of the membrane and the cytoplasm, and immunogenicity analysis of LppA protein.** The sequence of Tn inserts sites of *M. bovis*Δ*LppA* strain and the sequence of *M. bovis LppA* optimized with *E. coli*-preferred codons.

## Data Availability

The datasets analysed in the current study are available from the corresponding author on reasonable request.
