# Supplementary figures and images for: LppA is a novel plasminogen receptor of Mycoplasma bovis that contributes to adhesion by binding the host extracellular matrix and Annexin A2
Source: Vet Res. 2023 Nov 17;54:107. doi: 10.1186/s13567-023-01242-1 (PMC10657132; doi:10.1186/s13567-023-01242-1)

**A**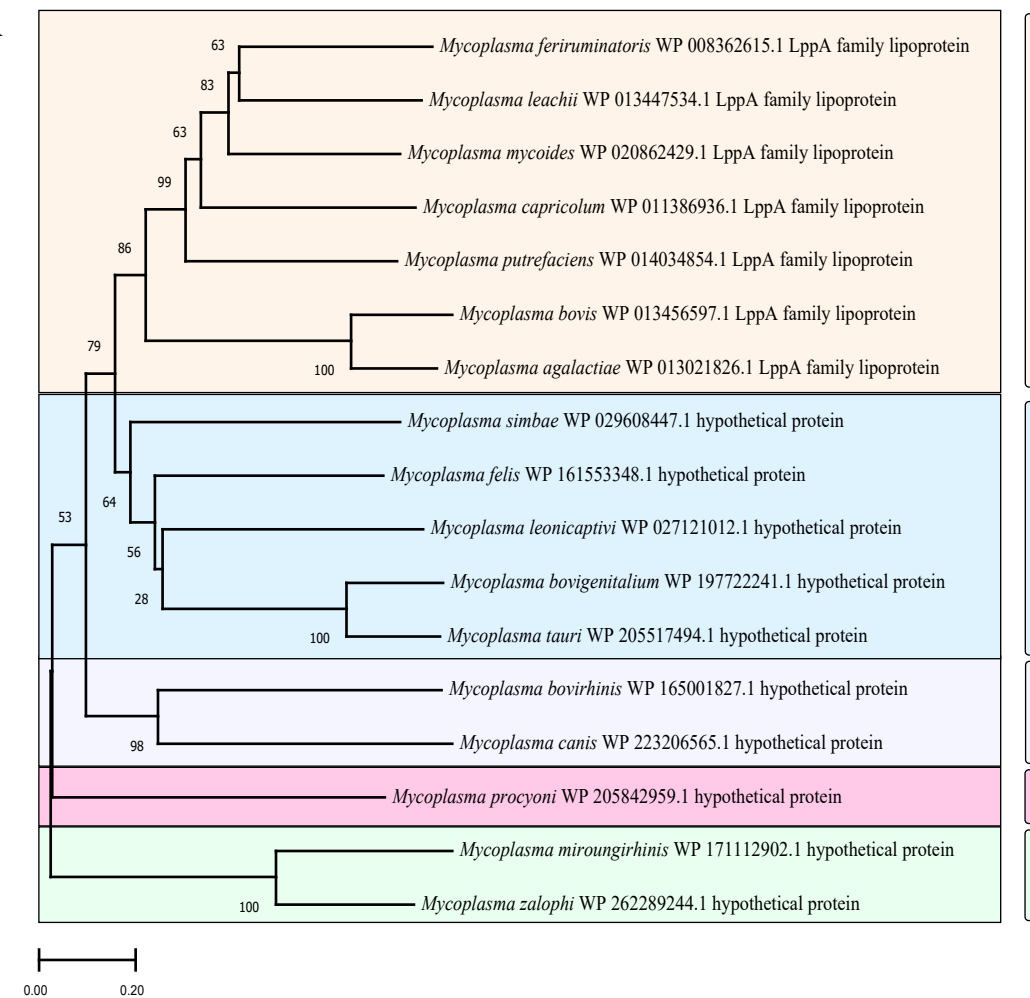**C**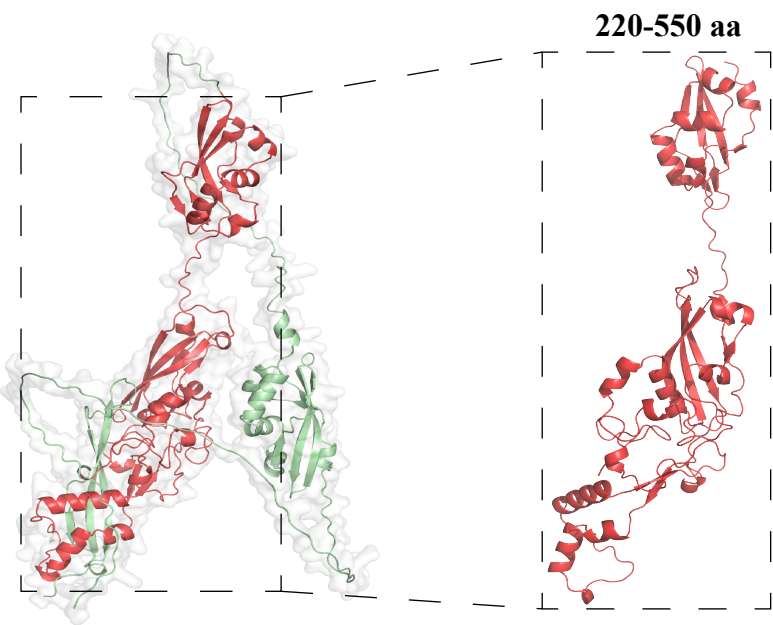**B**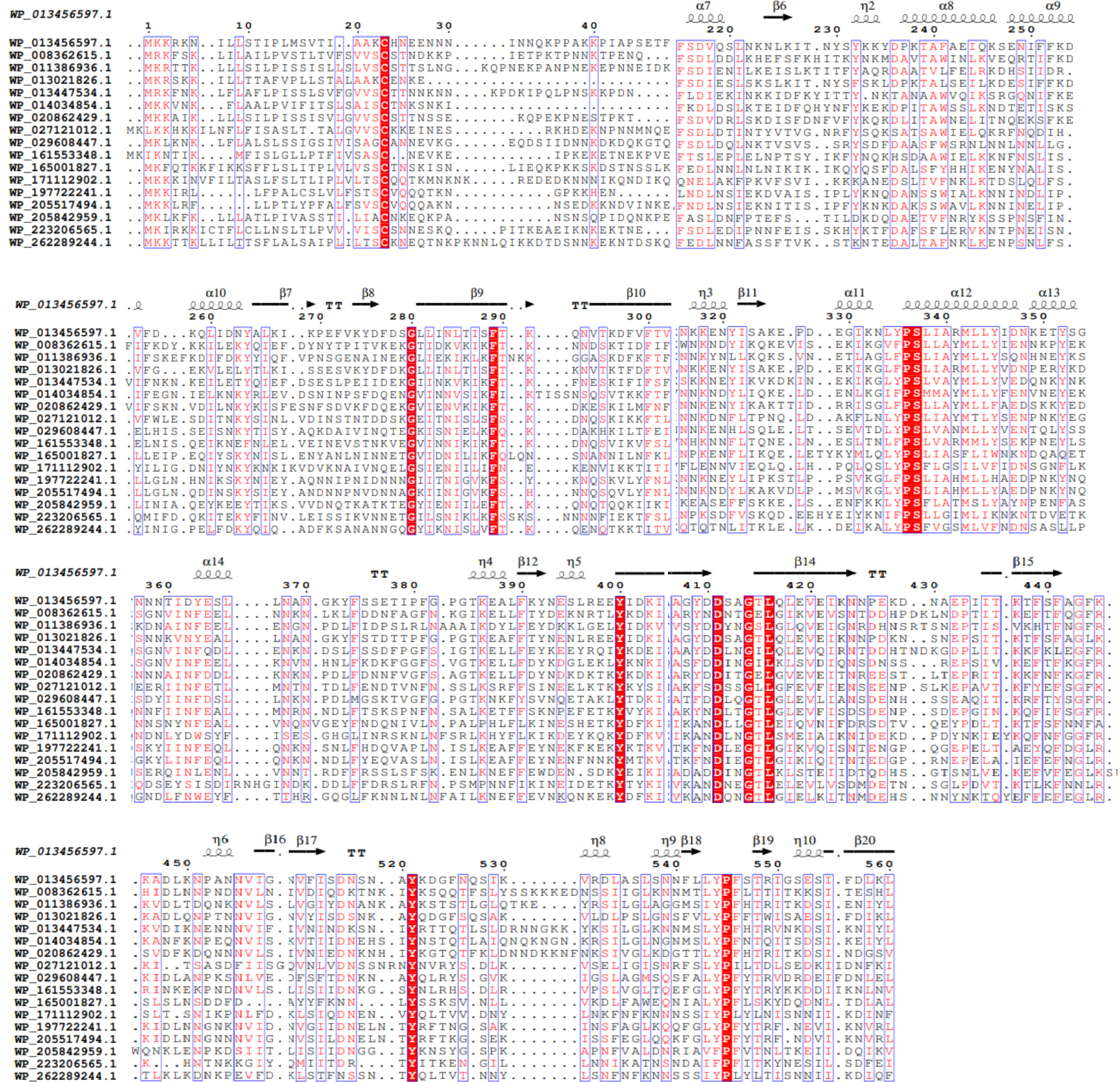

Supplement: Supplementary file 3 — Additional file 3. Sequence characterization of LppA in different species of the genus Mycoplasma. A. Phylogenetic tree constructed based on LppA sequences was divided into five groups based on the topological structure. Groups 1 to 5 are marked with yellow, blue, purple, pink, and green, respectively. The LppA of M. bovis and M. agalactiae clustered in Group 1. B. Multiple conserved sites of LppA in Mycoplasma were revealed. Highly conserved residues are shown in red. C. Protein structures of M. bovis LppA were modeled using AlphaFold2; red indicates the most conserved region. [file 13567_2023_1242_MOESM3_ESM.pdf]

**A**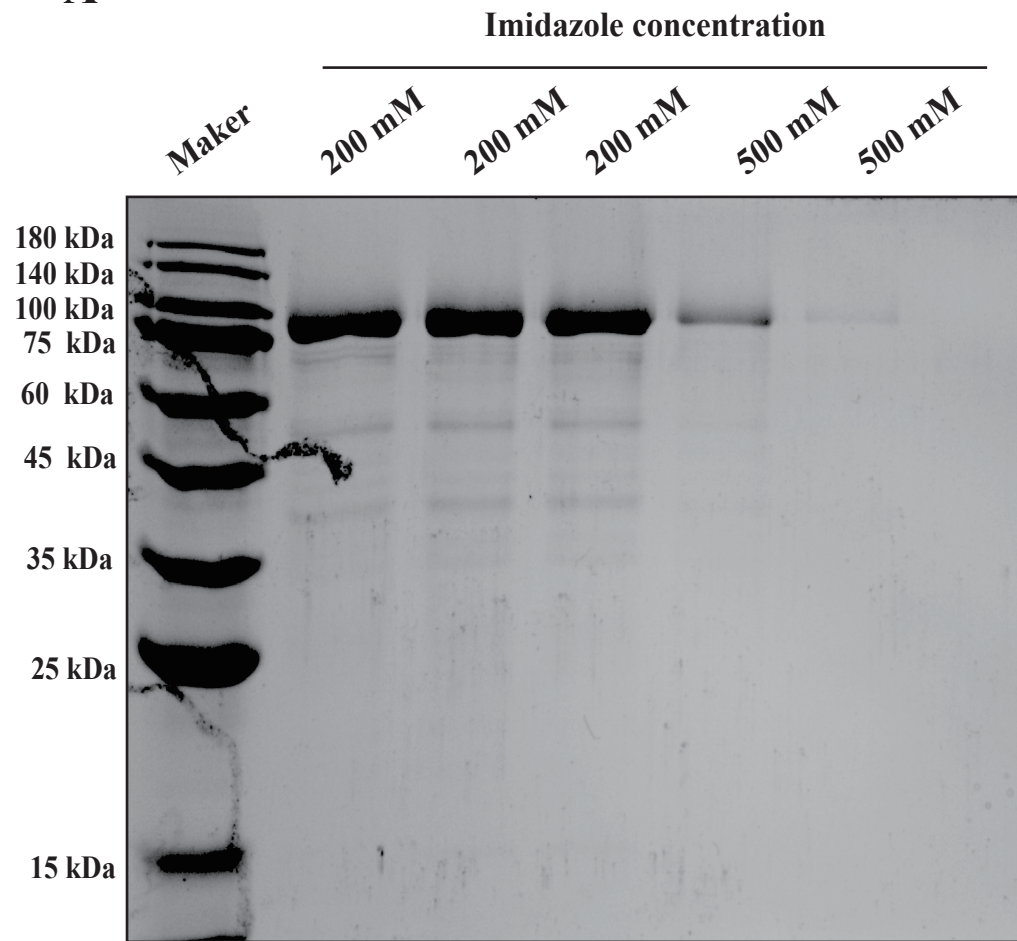**B**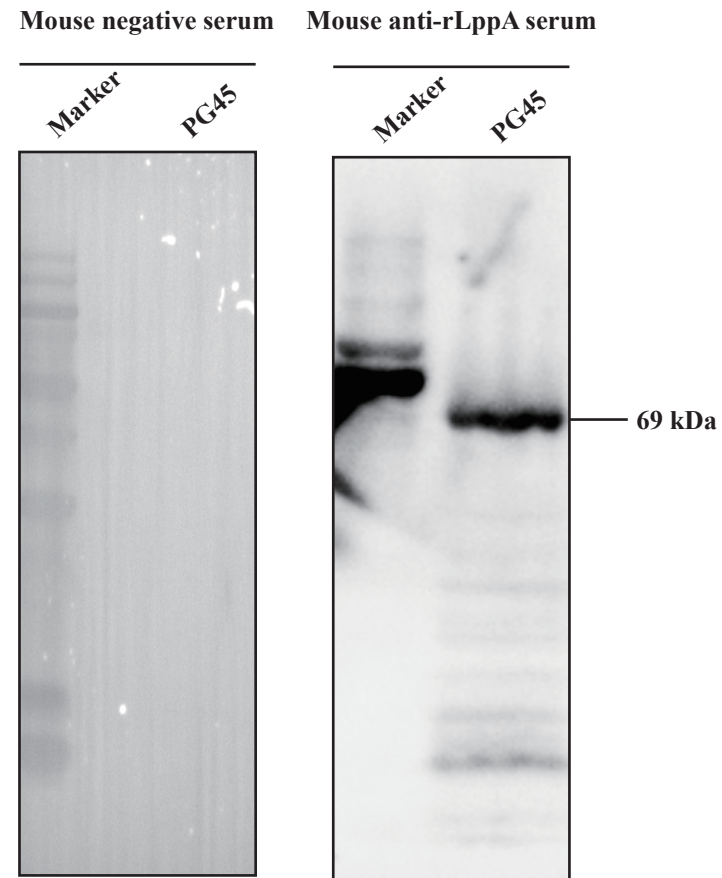

Supplement: Supplementary file 4 — Additional file 4. Recombinant LppA expression and polyclonal antibody preparation. A. Purification of the rLppA protein washed with different concentrations of imidazole solution and analyzed using sodium dodecyl sulfate–polyacrylamide gel electrophoresis. B. LppA of M. bovis PG45 was examined by western blot using anti-LppA mouse serum and negative serum. [file 13567_2023_1242_MOESM4_ESM.pdf]

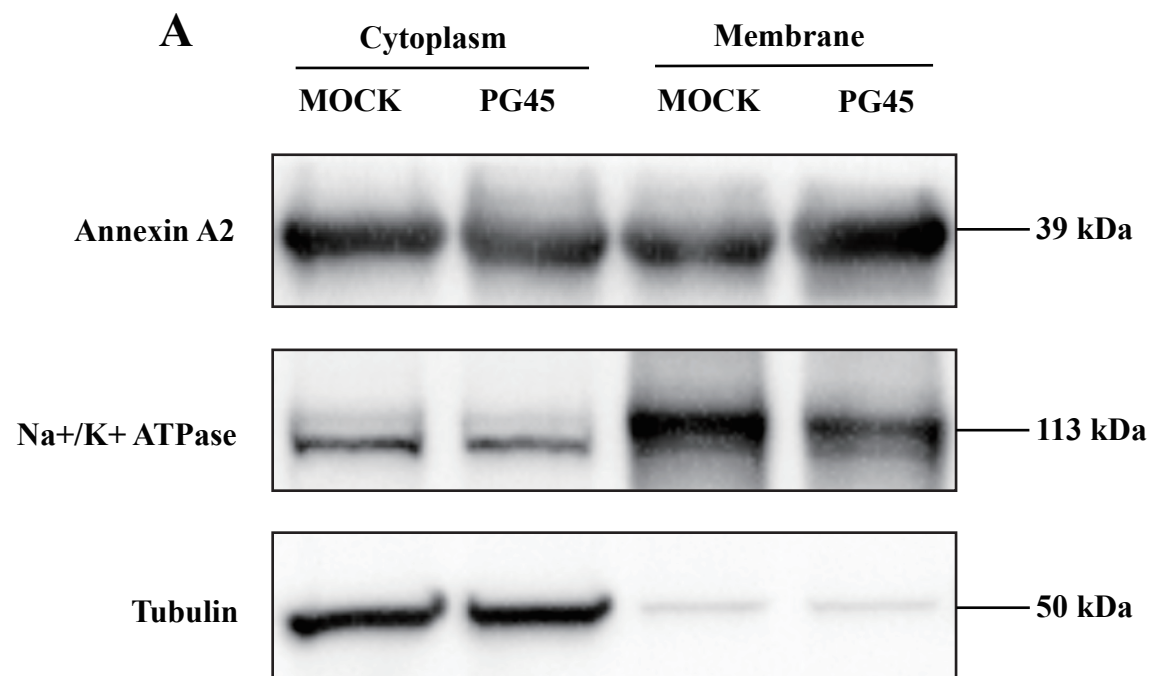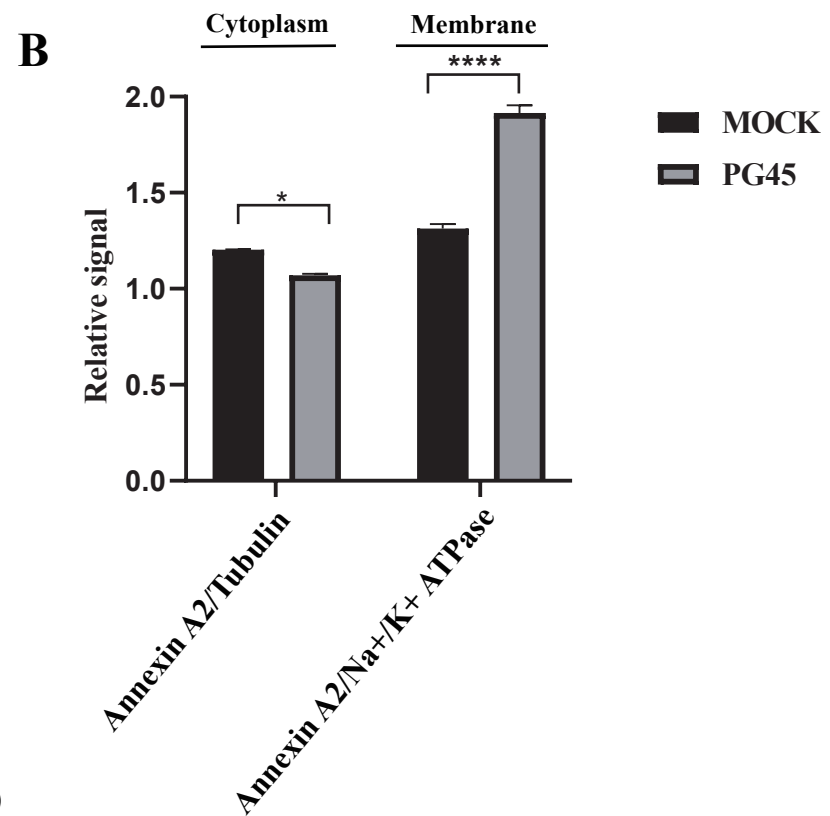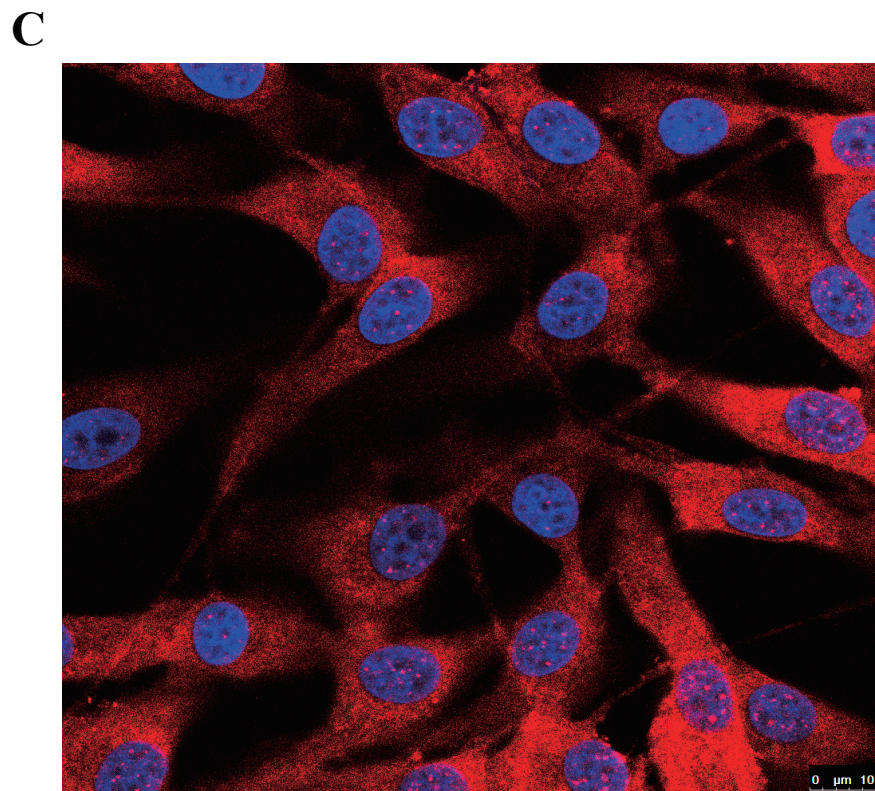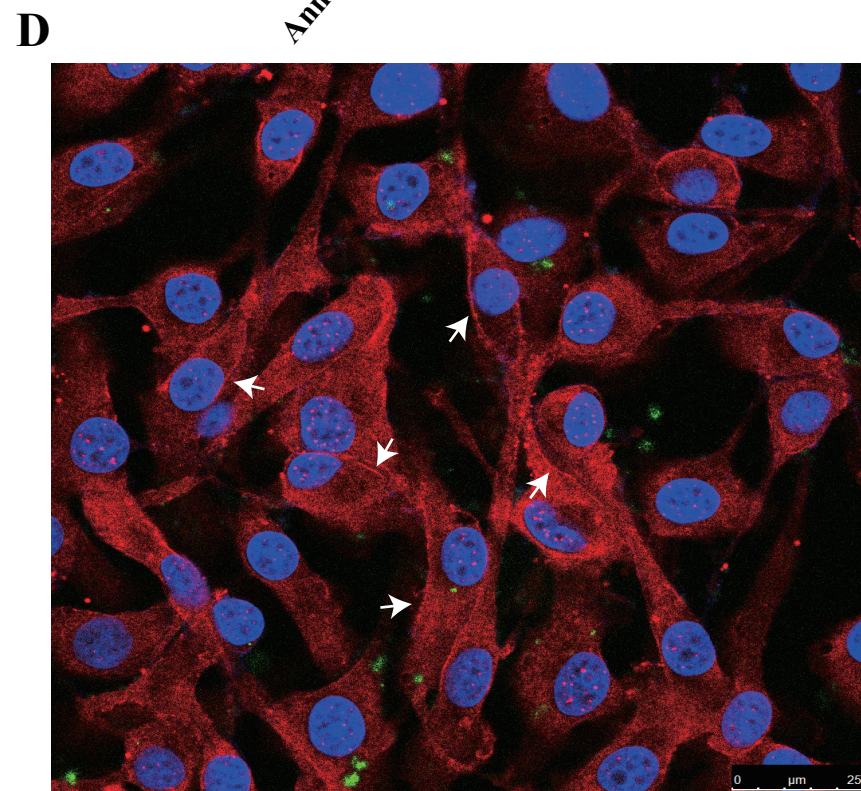

Supplement: Supplementary file 6 — Additional file 6. M. bovis infection promotes ANXA2 translocation from the cytoplasm to the membrane. The embryonic bovine lung (EBL) cells were infected with M. bovis PG45 for 24 h. A. Expression changes of ANXA2 in the cell membrane and cytoplasm were detected by using an anti-ANXA2 antibody. α-Tubulin and Na + /K + -ATPase were used as marker proteins for the cell cytoplasm and membrane, respectively. B. Relative ANXA2 expression in the cell membrane and cytoplasm. C. EBL cells uninfected with the M. bovis PG45. D. EBL cells infected with the M. bovis PG45. Blue represents DAPI, red represents ANXA2, and green represents M. bovis. The white arrow indicates ANXA2 translocation from the cytoplasm to the membrane. [file 13567_2023_1242_MOESM6_ESM.pdf]
