# Supplementary material for: LppA is a novel plasminogen receptor of Mycoplasma bovis that contributes to adhesion by binding the host extracellular matrix and Annexin A2
Source: Vet Res. 2023 Nov 17;54:107. doi: 10.1186/s13567-023-01242-1 (PMC10657132; doi:10.1186/s13567-023-01242-1)
Supplement: Supplementary file 5 — Additional file 5. Immunogenicity analysis of LppA in M. bovis. A. rLppA protein was examined using western blot using different sera. M. bovis-negative bovine serum was used as the negative control. B. Presence of LppA was examined in six strains of M. bovis by using anti-LppA serum. [file 13567_2023_1242_MOESM5_ESM.pdf]

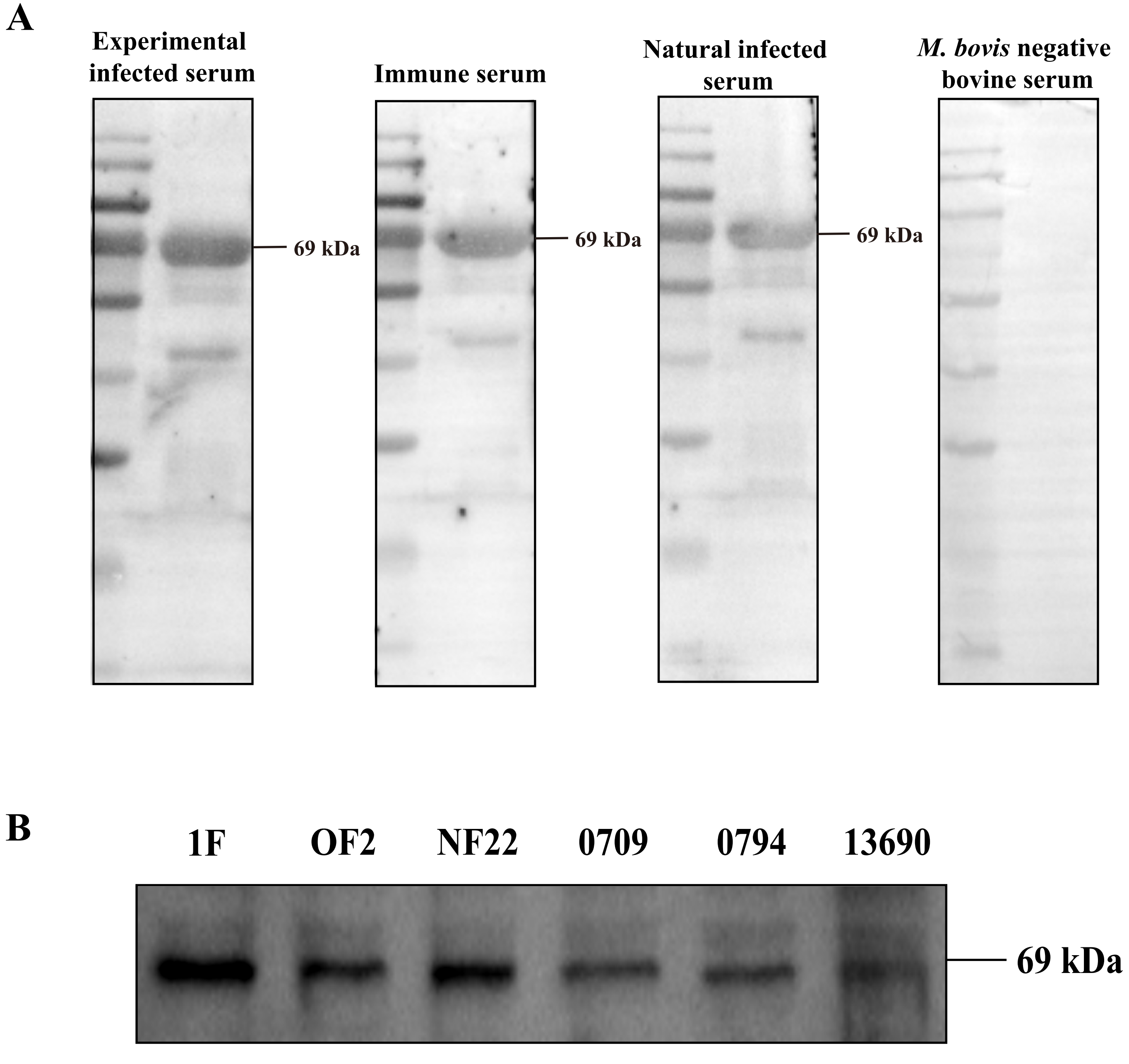
A
Experimental infected serum
Immune serum
Natural infected serum
M. bovis negative bovine serum
69 kDa
69 kDa
69 kDa
B
1F
OF2
NF22
0709
0794
13690
69 kDa
