# Supplementary material for: LppA is a novel plasminogen receptor of Mycoplasma bovis that contributes to adhesion by binding the host extracellular matrix and Annexin A2
Source: Vet Res. 2023 Nov 17;54:107. doi: 10.1186/s13567-023-01242-1 (PMC10657132; doi:10.1186/s13567-023-01242-1)
Supplement: Supplementary file 7 — Additional file 7. The methods of construction and identification of M. bovis PG45 mutant strains, expression and purification of the recombinant protein, extraction of the membrane and the cytoplasm, and immunogenicity analysis of LppA protein. The sequence of Tn inserts sites of M. bovisΔLppA strain and the sequence of M. bovis LppA optimized with E. coli-preferred codons. [file 13567_2023_1242_MOESM7_ESM.docx]

**Additional file 7 Additional material**

**Methods**

**Construction and identification of *M. bovis* PG45 mutant strains**

*M. bovis* mutant strains were constructed by transforming the pMT85-derived plasmid (pMT/mNeonGreen) using the PEG method. Briefly, *M. bovis* PG45 was cultured to the exponential phase, washed twice with Dulbecco’s phosphate-buffered saline, and then suspended in 0.1 M CaCl_2_. *M. bovis* was cultured in a medium containing 20 μg of plasmid DNA, 10 μg of yeast tRNA, and 70% PEG8000. Following this, the cells were centrifuged, incubated in a pleuropneumonia-like organism (PPLO) medium, and plated on PPLO solid medium with the required kanamycin antibiotic (100 μg/mL) at 37 °C under 5% CO_2_. Single colonies on agar were confirmed by polymerase chain reaction and sequencing. The ΔLppA mutants were identified using the LppA-F/R primer (Additional file 1).

**Expression and purification of the recombinant protein**

The plasmid pET30a-LppA was transformed into *E. coli* BL21 (DE3) for recombinant protein expression. Transformants were cultured in Luria Broth (LB) medium containing 100 μg/mL kanamycin at 37 °C for 16 h, then transferred into fresh LB medium containing 100 μg/mL kanamycin at a 1:10 ratio and allowed to grow at 37 °C with shaking until the optical density at 600 nm reached 0.6. IPTG (0.5 mM) was then added to the cultures to induce recombinant protein expression. The IPTG-induced bacteria were pelleted at 8000 rpm for 20 min at 4 °C, resuspended in ice-cold phosphate-buffered saline buffer, and disrupted by sonication at 4 °C for 10 min (5 s on, 10 s off, for 40 cycles). After centrifugation, the supernatant was filtered through a 0.22-μm membrane to remove cell debris. The resultant supernatant was loaded onto Ni–NTA agarose resin (Qiagen) for protein purification. Protein was eluted with a column buffer containing 500 mM imidazole and then concentrated. Imidazole was removed from the recombinant protein fractions using ultra-centrifugal filters with a 10 kDa cutoff (Millipore Sigma). Purified recombinant proteins were stored at −80 °C until used.

**Extraction of the membrane and the cytoplasm**

A commercial membrane protein extraction kit (Thermo Fisher Scientific) was used to separate cell membrane and cytoplasmic proteins according to the manufacturer's instructions. Briefly, embryonic bovine lung (EBL) cells or *M. bovis* PG45 were centrifuged at 300 × *g* (10 000 × *g* for *M. bovis*) for 5 min, washed twice with cell wash solution, and the supernatant was carefully removed and discarded. The EBL cells or *M. bovis* PG45 were resuspended in the cell wash Solution, then centrifuged for 5 min at 300 × *g*, and the supernatant was discarded. The cell pellet was resuspended in 0.75 mL of permeabilization buffer, briefly vortexed, and incubated for 10 min at 4 °C with constant mixing. The permeabilized cells were centrifuged for 15 min at 16 000 × *g*. The supernatant containing cytosolic proteins was transferred to a new tube and stored for analysis. Then, 0.5 mL of solubilization buffer was added to the pellet, and the cells were resuspended by pipetting up and down. The tubes were incubated at 4 °C for 30 min with constant mixing and then centrifuged at 16 000 × *g* for 15 min at 4 °C. The supernatant containing solubilized membrane and membrane-associated proteins was transferred to a new tube. Both membrane and cytoplasm fractions could be used immediately or stored at −80 °C for future use.

**Immunogenicity analysis of LppA protein**

rLppA was transferred to a polyvinylidene fluoride (PVDF) membrane; the membrane was incubated with *M. bovis* negative bovine serum (1:100 dilution), immune serum (1:100 dilution) from mice immunized with rLppA protein, infected serum (1:100 dilution) from calves experimentally infected with *M. bovis*, and pooled serum (1:100 dilution) from calves naturally infected with *M. bovis*. It was then probed with horseradish peroxidase (HRP)-conjugated goat antibovine antibody (1:2000 dilution) or HRP-conjugated goat anti-mouse antibody (1:5000 dilution). The immunogenicity of LppA in different *M. bovis* strains was analyzed. Whole-cell proteins of PG45 and six other *M. bovis* strains were separated by sodium dodecyl sulfate–polyacrylamide gel electrophoresis, transferred onto a PVDF membrane, incubated with a 1:1000 dilution of mouse anti-LppA serum, and then probed with a 1:5000 dilution of HRP-conjugated goat antimouse antibody.

**Results**

**Sequence of Tn insert sites of *M. bovis*Δ*LppA* strain**

GTGACCCTTGAAAACGAGCGCAGCGAGTCAGTGAGCGAGGAGCGGAAGAGCGCCCAATACGCAAACCGCCTCTCCCCGCGCGTTGGCCGATTCATTAATGCACGCTAGCGGATCTCATAAAAATGTATCCTAAATCAAATATCGGACAAGCAGTGTCTGTTATAACAAAAAATCGATTTAATAGACACATTAACAGCACTGTTTTTATGTGTGCGATAATTTATAATATTTCGGACGGTTGCGGTACCCTTTTACACAATTATACGGACTTTATCCATAATTATCTATTAATTGCTTGTCAAAAACGTCTTTAAAAAAGATGTTTTCGCTTTTTTGTATTTCAGCAAAAGCAGTTTTTGGATCGTACTTTTTATATGAATAATTTGTTATTTTCAAATTCTTATTTAATGATTGTACATCGCTAAAATCATCGTTTTTAGTATTTGTGTTTTCATTATTGTCAGGCTCAGTGCGTTCAACATCTGGTCTCCTTGGCTCAAATGTCTCTGACGGGGCAATAGGTTTTTTTGCAGGCGGTTTCTGATTATTTATATTGTTGTTTTCTTCATTATGACACTTAGCAGCTATAGTGACACTCATTAATGGAATAGTAGATAATAAAATATTTTTCCTTTTCTTCATAATAAACCTCTGACAAAATAATTCTATATTTTTATATAGCATAATCAAAAAATAATTCATTACTTTGACCCAATAATACTGTTTTAAAGCCTTTTTGGAATTATTCAGTTAAATTGATAATGTTTCATAATTACTATGACCTTCAACCTTGATGCCACAAAATTATTTATTTTAATAAATAATCTGCCTTGATGCTTTCTCTAATTTGCTAATAAAAATATAATAAGCTTATGAATGAATTTATAGATTTATACAACACAACTGAACATTCATTTTTAGAATCATTGATCAAGGGTTAAAGATCTAGTTAGATTATCGAAAAAAAATGGCAAAAAAAGGCCGGTTGTTTTAAGTGATCATAATATCTATTTGCATTAGGGTGAATTTTTAAAACAATGCAATATTTTATGACATTTAACCCAATAATTGAATGAAATTATCGTGAATCAGGTGCTCTTATAATGTTTTGTATGAGACCTGATGCATTTGATGCTTATTTGCAGATTGCACAGTTGGAATGACATCCATACGGACGGCTAGTTACGATGCCTAACCAAATGGAAGGAAT

**Sequence map of Tn insert sites of *M. bovis*Δ*LppA* strain**


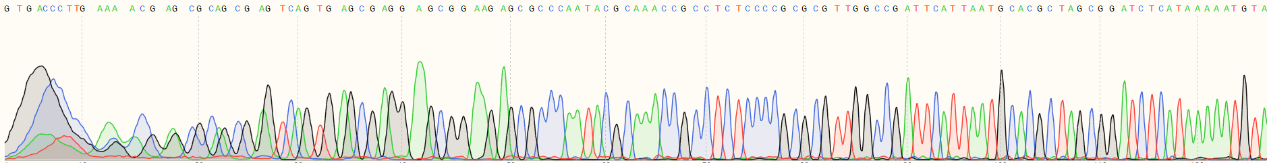


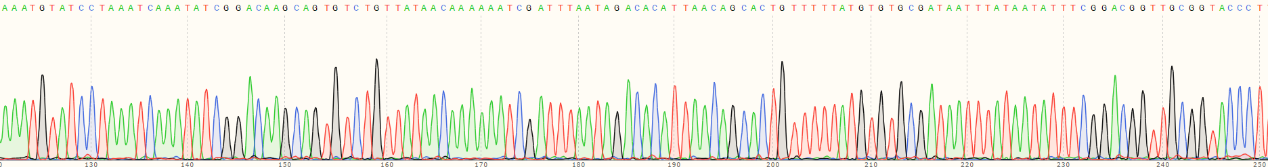


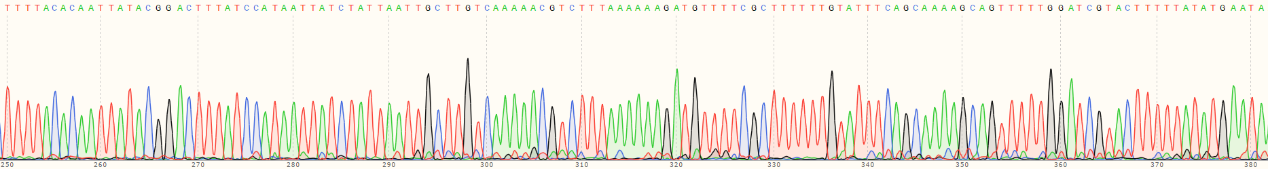


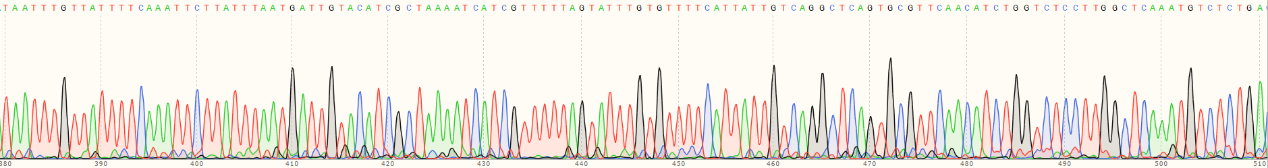


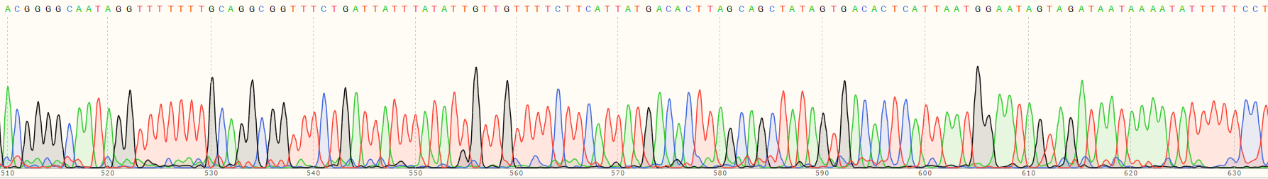


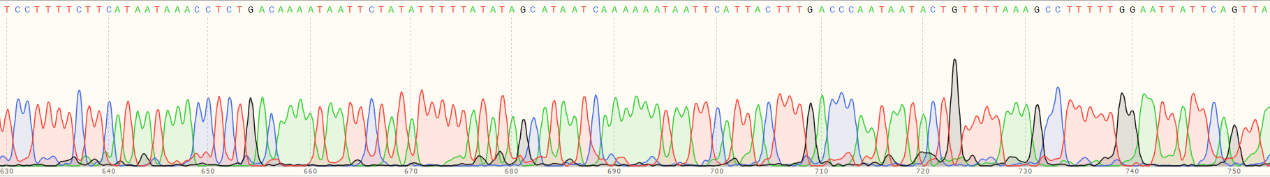


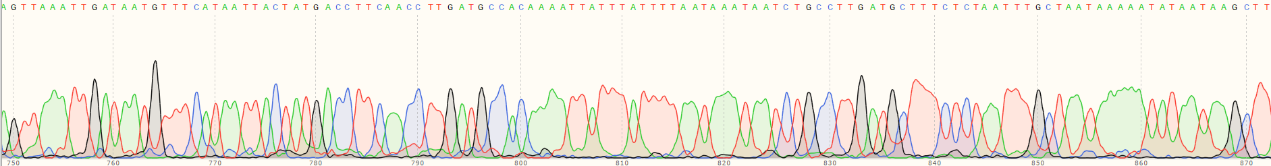


**The full sequence of *M. bovis* LppA was optimized with *E. coli*-preferred codons**

gaattcATGAGCGTGACCATCGCGGCGAAATGCCACAACGAAGAAAACAACAACATCAACAACCAGAAACCGCCAGCGAAAAAACCGATCGCGCCGTCTGAAACCTTCGAACCGCGCCGTCCGGACGTGGAACGCACCGAACCGGATAACAATGAAAACACCAACACTAAAAACGACGACTTTTCTGACGTTCAGAGCCTGAACAAAAACCTGAAAATTACCAACTACAGCTACAAAAAATACGACCCGAAAACCGCTTTCGCGGAAATCCAGAAAAGCGAAAACATCTTTTTCAAAGATGTATTCGATAAACAGCTGATCGATAACTACGCGCTGAAACTGAAACCGGAGTTCGTTAAATATGATTTTGACTCTGGTCTGCTGATCAACCTGACCATCTCCTTCACCAAACAGAACGTTACTAAAGATTTCGTTTTCACCGTGCATGGTTTTAAAAAAGCAGAACAGATCATCAACAACATCAACAATGAAAAACCGCCGGCGAAAAAACTGATCGCGCCATCCGAAACCTTCGAACCGCGTCGTCCGGATGTTGGCCGTACGGATCCGGACAACAACGAAAACACGAACACCAAAAACGTCGATTTCTCTGACGTTCAGTCCCTGAACAAAAACCTGAAAATCACCAACTATAGCTACAAAAAATACGATCCGAAAACCGCGTTCGCCGAAATCCAGAAATCTGAAAACATCTTCTTCAAAGATGTTTTCGACAAACAGCTGATCGACAACTATGCGCTGAAAATCAAACCGGAGTTCGTCAAATACGATTTCGACAGCGGCCTGCTGATCAACCTGACCATCTCTTTCACCAAACAGAACGTTACCAAGGATTTCGTTTTCACCGTACACGGCTTCAAGAAAACCGAACAGATCATCAACAACAAAAACAAAAAAGAGAACTACATCTCCGCGAAAGAACCGGATGAAGGTATTAAAAACTTATACCCGAGCCTGATCGCACGTATGCTGCTGTACATCGATAACAAAGAAACCTACTCTGGCATTGTGAACAACAACACCATCGACTACGAATCCCTGCTGAACGCGAACGGTAAATACTTTTCTTCTGAAACCATTCCGTTCGGTCCGGGCACCAAAGAAGCCCTGTTCAAATATAACGAAAGCCTGCGCGAAGAATACATCGACAAAATTATCGCTGCTGGCTACGACGACAGCGCCGGCACGCTGCAGCTGGAAGTGGAAATCAAAAACAACCCGGAAAAAGACAACGCGGAACCGATCATTACCAAGACCTTCAGCTTTGCGGGCTTCAAAAAAGCGGACCTGAAAAACCCGGCGAACAACGTTATTGGCTTTTTCCTGACCACCATGTCCTTCAAAAACCTGCAGGTGATCAAGAGCATCTTCTCTAAAACCACCAAAGAAAGCATCAAAGCAGGCCAGAACATCCTGAAAGATATTGATAAAAACAAAGCCGACTTCCTGAAACAGCGCATCATCAGCGAACTGAACGTTTTCATCTCCGACAACTCCAACGCGTACAAAGATGGTTTTAACCAGTCTATCAAAGTCCGCGACCTGGCGAGCCTGAGCAACAACTTCCTGCTGTACCCGTTCAGCACTCGTATTGGCAGCGAATCTATCTTCGACCTGAAACTGGAGCTGATTAACAACGACGGCGATAAACTGCGCCTGTCCTTTAACATCAAACTGCCGATCTTTGCACAGGGTATCGGCGACCTGAAAGATCACTCTGATAGCAACAACAAAGAAATCCTGATCCCGGTGAGCGTGGAAACCTACATCGACCAGTTCAGCTTCAAAAAATCTTAActcgag
